# Analysis of clinical distribution and drug resistance of klebsiella pneumoniae pulmonary infection in patients with hypertensive intra cerebral hemorrhage after minimally invasive surgery

**DOI:** 10.12669/pjms.38.1.4439

**Published:** 2022

**Authors:** Wei Li, Li Xu, Haige Zhao, Shanshan Zhu

**Affiliations:** 1 Wei Li, Clinical Laboratory, Affiliated Hospital of Hebei University, Baoding, 071000, Hebei, China; 2 Li Xu, Clinical Laboratory, Baoding Children’s Hospital, Baoding, 071000, Hebei, China; 3 Haige Zhao, Clinical Laboratory, Affiliated Hospital of Hebei University, Baoding, 071000, Hebei, China; 4 Shanshan Zhu, Clinical Laboratory, Affiliated Hospital of Hebei University, Baoding, 071000, Hebei, China

**Keywords:** Hypertensive intracerebral hemorrhage, lung, Klebsiella pneumoniae, clinical distribution, drug resistance

## Abstract

**Objectives:**

To investigate the clinical distribution and drug resistance of Klebsiella pneumoniae pulmonary infection in patients with hypertensive intracerebral hemorrhage after minimally invasive surgery.

**Methods:**

A total of 658 patients with hypertensive intracerebral hemorrhage who underwent minimally invasive surgery admitted to the intensive care unit (ICU) and the Department of Neurology of Affiliated Hospital of Hebei University from January 2015 to January 2020 were enrolled and divided into two groups: the observation group and the control group. Three hundred and thirty-three cases with postoperative pulmonary infection were included into the observation group, and 325 cases without postoperative pulmonary infection were divided into the control group. The intubation time, neurological deficiency score and Glasgow coma scale (GCS) of the two groups were analyzed and compared. Automatic microbial identification system was utilized to isolate bacteria from patients in the observation group, identify Klebsiella pneumoniae, and analyze Klebsiella pneumoniae infection, clinical department distribution, and age distribution. The Kirby-Bauer method was adopted to carry out the drug susceptibility test of Klebsiella pneumoniae infection.

**Results:**

The intubation time and neurological deficiency score of patients with hypertensive cerebral hemorrhage in the observation group were significantly higher than those in the control group (*p*<0.05), while the GCS score was significantly lower than that in the control group *(p*<0.05). A total of 403 strains of pathogenic bacteria were isolated from 325 patients in the observation group, of which 52 strains of Klebsiella pneumoniae were detected in 52 patients with postoperative pulmonary infection, accounting for 12.90%. The detection rates of Klebsiella pneumoniae in ICU and neurology department were 53.85% and 46.15%, respectively. Klebsiella pneumoniae had the highest detection rate (40.38%) in people aged 70 years and above. Moreover, fifty-two strains of Klebsiella pneumoniae showed low drug resistance rate (<20%) to cefoperazone/sulbactam, piperacillin/tazobactam, cefoxitin, imipenem, meropenem, amikacin, ciprofloxacin, and levofloxacin.

**Conclusion:**

For patients with hypertensive cerebral hemorrhage who have pulmonary infection after minimally invasive surgery, risk factors causing infection should be identified in time, their Klebsiella pneumoniae infection should be correctly monitored, and antibiotics should be taken rationally to effectively promote the elimination of brain edema in patients and protect the cranial nerve function of patients.

## INTRODUCTION

Hypertension, as one of the clinically common chronic diseases, is prone to cause cardiovascular and cerebral vascular diseases^1^. In recent years, the incidence of hypertensive intracerebral hemorrhage has been hovering at a high level driven by a high incidence of hypertension^2^. Hypertensive intracerebral hemorrhage falls into a critical acute cerebrovascular disease whose bleeding volume and bleeding site have a close bearing on the prognosis of patients. Patients with hypertensive intracerebral hemorrhage may be directly life-threatening if the bleeding site is located in the brainstem and the amount of bleeding is large^3^,^4^

Previous clinical studies have shown that most patients with hypertensive intracerebral hemorrhage after minimally invasive surgery are prone to pulmonary infection induced by various factors such as coma, pulmonary aspiration, long bed stay, resulting in the deterioration of the patient’s condition, or even the death of patients[5]. Klebsiella pneumoniae is a critical pathogenic bacteria with drug resistance that can give rise to clinical infections. Infections caused by Klebsiella pneumoniae may lead to local outbreaks in hospitals, posing great challenges to clinical treatment[6]. For the purpose of further analyzing the clinical distribution and drug resistance of Klebsiella pneumoniae pulmonary infection in patients with hypertensive intracerebral hemorrhage after minimally invasive surgery, in this study, patients with hypertensive intracerebral hemorrhage admitted to the ICU and the Department of Neurology of our hospital were enrolled as research objects.

## METHODS

A total of 333 patients with hypertensive intracerebral hemorrhage who underwent minimally invasive surgery admitted to the intensive care unit (ICU) and the Department of Neurology of Affiliated Hospital of Hebei University from January 2015 to January 2020 were selected as the observation group. It included, 168 males and 165 females, aged 43-81 years, with an average age of (65.08±14.96) years old. There were 186 cases of basal ganglia hemorrhage, 67 cases of thalamus hemorrhage, 53 cases of cerebellar hemorrhage, and 27 cases of brain stem hemorrhage. In addition, 325 patients with hypertensive cerebral hemorrhage without pulmonary infection admitted to Affiliated Hospital of Hebei University during the same period of time were selected as the control group, including 166 males and 159 females; aged 42-80 years, with an average age of (65.14±15.02) years old. There were 180 cases of basal ganglia hemorrhage, 66 cases of thalamic hemorrhage, 52 cases of cerebellar hemorrhage, and 27 cases of brainstem hemorrhage. There was no statistically significant difference in general information between the two groups (*p*>0.05), which were comparable.

### Ethical Approval:

The study was approved by the Institutional Ethics Committee of Affiliated Hospital of Hebei University(No.20190924; date: April 2018), and written informed consent was obtained from all participants

Reference was made to the clinical criteria for diagnosis and treatment of hypertensive intracerebral hemorrhage in the “Guidelines for Diagnosis and Treatment of Intracerebral Hemorrhage in China” (2014)^7^

### Inclusion criteria:

1. Patients who meet the relevant diagnostic criteria for hypertensive intracerebral hemorrhage;
2. Patients with a history of hypertension;
3. Patients who underwent minimally invasive intracranial hematoma removal under local anesthesia within 4-12h after admission. Pulmonary infection criteria: The diagnosis was made according to the criteria for pulmonary infection in the “Guidelines for the Diagnosis and Treatment of Infectious Diseases”^8^. Inclusion criteria for patients with pulmonary infection after minimally invasive surgery for hypertensive intracerebral hemorrhage: Patients with hypertensive intracerebral hemorrhage who had postoperative pulmonary infection all met the criteria for pulmonary infection and were confirmed by CT examination.

### Exclusion criteria:

1. Patients with incomplete clinical examination data;
2. Patients who withdrew from the study halfway.

Antimicrobial susceptibility disk (purchased from Oxoid, UK), Mueller-Hinton medium for drug sensitivity (self-prepared). Isolation and identification of Klebsiella pneumoniae. Deep sputum samples were taken from patients in the observation group, and Klebsiella pneumoniae was isolated and identified using an automatic microbial identification system.

After minimally invasive surgery, penicillin/cephalosporin antibiotics were used for the prevention of pulmonary infection in all patients with hypertensive intracerebral hemorrhage, and levofloxacin quinolone antibiotics were selected for the prevention of pulmonary infection in patients with a history of penicillin drug allergy. After the diagnosis of patients with pulmonary infection, medication can be carried out according to previous empirical standards, and then the use of antibiotics can be adjusted according to the clinical symptoms of patients until the bacterial report of pulmonary infection in patients. Patients without pulmonary infection after minimally invasive intracerebral hemorrhage received general routine treatment. The endotracheal intubation time, neurological deficiency and GCS score were observed in both groups. Among them, neurological deficiency and GCS were evaluated according to neurological deficiency score criteria and GCS assessment criteria, respectively. The Kirby-Bauer method recommended by CLSI was used for drug susceptibility tests^9^, and the results were interpreted strictly in accordance with the applicable standards established by CLSI. Klebsiella pneumoniae ATCC700603 was used as the quality control bacteria.

### Statistical Analysis:

All the experimental data of this study were analyzed using SPSS22.0 statistical software, and the measurement data were expressed as mean ± standard deviation (±s). Comparison between groups was performed by the independent sample t test, the count data was expressed in percentage or constituent ratio (%), and the chi-square test was adopted for intra-group pairing. P<0.05 indicates a statistically significant difference.

## RESULTS

Compared with the control group, the intubation time and neurological function defect scale of patients with hypertensive intracerebral hemorrhage in the observation group were significantly increased (*p*<0.05), and the GCS score was significantly decreased (*p*<0.05). Table-I A total of 403 strains of pathogenic bacteria were isolated from 333 patients with hypertensive intracerebral hemorrhage complicated with pulmonary infection, of which 52 strains of Klebsiella pneumoniae were detected in 52 patients with hypertensive intracerebral hemorrhage complicated with pulmonary infection, accounting for 12.90%.

**Table-I:** Comparison of intubation time, neurological function defect scale and GCS score between the two groups.

Group	Number of cases	Intubation time (h)	Neurological deficiency score (points)	GCS score (points)
Control group	325	18.10±4.32	17.08±3.31	8.94±1.30
Observation group	333	76.69±15.23	23.87±5.48	1.56±0.45
t		66.780	19.182	97.768
p		<0.001	<0.001	<0.001

Among 52 patients with hypertensive intracerebral hemorrhage who had Klebsiella pneumoniae pulmonary infection after minimally invasive surgery, 28 cases were in ICU and 24 cases were in the Neurology Department, with their constituent ratios of 53.85% and 46.15%, respectively. Seven cases (13.46%) were isolated from populations aged <40 years, six cases (11.54%) from those aged 40-49 years, eight cases (15.38%) from those aged 50-59 years, ten cases (19.23%) from those aged 60-69 years, and 21 cases (40.38%) from those aged >70 years.

The antibacterial drugs with high drug resistance rate of Klebsiella pneumoniae were ampicillin (96.30%), piperacillin (48.08%), cefazolin (44.23%) and sulfamethoxazole (42.31%), and the antibacterial drugs with low drug resistance rate of Klebsiella pneumoniae were imipenem (5.77%), meropenem (3.85%), amikacin (3.85%) and levofloxacin (3.85%), respectively. The antibacterial drugs with high sensitivity of Klebsiella pneumoniae were meropenem (94.23%), amikacin (92.31%), levofloxacin (92.31%) and imipenem (90.38%), respectively, while the antimicrobial drugs with the lowest sensitivity of Klebsiella pneumoniae was amphenicillin (1.92%). Table-II

**Table-II:** Drug resistance rate and sensitivity rate of Klebsiella pneumoniae to commonly used antibacterial drugs (%).

Antibacterial drugs	Drug resistance rate (%)	Sensitivity (%)
Ampicillin	96.30	1.92
Piperacillin	48.08	32.69
Amoxicillin/Clavulanate	23.08	57.69
Ampicillin/Sulbactam	28.85	67.31
Cefoperazone/Sulbactam	19.23	69.23
Ticacillin/Clavulanate	28.85	46.15
Piperacillin/Tazobactam	19.23	65.38
Cefazolin	44.23	26.92
Cefoperazone	38.46	59.62
Cefuroxime	34.62	63.46
Ceftriaxone	36.53	57.69
Ceftazidime	25.00	63.46
Cefotaxime	34.62	51.92
Cefepime	21.15	75.00
Cefoxitin	11.54	78.85
Aminotronan	28.84	65.38
Imipenem	5.77	90.38
Meropenem	3.85	94.23
Amikacin	3.85	92.31
Gentamicin	25.00	69.23
Ciprofloxacin	11.54	65.38
Levofloxacin	3.85	92.31
Compound Sulfamethoxazole	42.31	53.85

## DISCUSSION

Hypertensive intracerebral hemorrhage is an intracerebral hemorrhage caused by the rupture of cerebral arterioles accompanied by hypertension during the sudden rise of blood pressure, with clinical characteristics of rapid onset, high disability rate and high mortality.^10^ Patients suffering from hypertensive intracerebral hemorrhage are often accompanied by edema, hematoma and cerebral hernia due to cerebral hemorrhage. In such a case, the intracranial pressure of patients will continue to rise, hematoma stasis will appear, and brain tissue will be compressed, eventually leading to disability and even death of patients.^11^ Minimally invasive surgery is currently used clinically to remove hematoma timely, and control of intracranial pressure is the principal approach to the treatment of hypertensive intracerebral hemorrhage.^12^ Pulmonary infection, as a frequent complication after minimally invasive surgery for patients with hypertensive intracerebral hemorrhage, has a serious adverse effect on postoperative recovery of patients.^13^ Consequently, it is of profound significance to search for pathogenic bacteria related to pulmonary infection to improve the prognosis of patients.

Patients with hypertensive intracerebral hemorrhage are often complicated with pulmonary infection after minimally invasive surgery, which seriously affects their mental state, neurological deficiency and quality of life.^14^ It has been reported in relevant literature that patients with hypertensive intracerebral hemorrhage worsen with the severity of the neurological deficiency.^15^ It was further shown in other studies that patients with hypertensive intracerebral hemorrhage are prone to respiratory infections after surgery, and Klebsiella pneumoniae is the principal pathogenic bacteria isolated. Preoperative low GCS score is a risk factor for postoperative infection in patients, and careful attention should be paid to the postoperative condition of patients with hypertensive intracerebral hemorrhage to prevent postoperative infection and exacerbation of the disease.^16^ In this study, the intubation time and neurological deficiency score of patients with hypertensive intracerebral hemorrhage in the observation group are significantly higher than those of the control group, and the GCS score is significantly lower than that of the control group, suggesting that patients with hypertensive intracerebral hemorrhage complicated with pulmonary infection may have problems such as longer intubation time, severe neurological deficiency, and too low GCS score. To this end, postoperative complications should be strictly monitored in clinical practice, and the degree of neurological deficiency and GCS score should be evaluated in time to prevent patients from getting worse or even dying.

Klebsiella pneumoniae, as the most common pathogen of nosocomial infections, may cause infections in a variety of parts of the respiratory tract and digestive tract. It is widely distributed in natural environments such as water and soil, and is easily colonized in the respiratory and gastrointestinal tracts of hospitalized patients.^17^ Studies have reported that Klebsiella pneumoniae has a high proportion of pathogenic bacteria in patients with hypertensive intracerebral hemorrhage complicated with pulmonary infection, accounting for 16.48%.^18^ It was also found in the study of Ji X H^19^ that Klebsiella pneumoniae accounted for 15.79% of the pathogenic bacteria in middle-aged and elderly patients with hypertensive basal ganglia cerebral hemorrhage complicated by pneumonia. In this study, Klebsiella pneumoniae accounts for 12.90% of the pathogenic bacteria in patients with hypertensive intracerebral hemorrhage complicated with pulmonary infection, which is slightly different from the proportion mentioned in previous studies. Such a difference may have a bearing on regional differences, suggesting that the awareness of nosocomial infection of medical staff should be strengthened, invasive operations should be reduced, indoor ventilation should be conducted, effective disinfection and isolation measures should be taken to avoid cross infection and prevent the epidemic of Klebsiella pneumoniae infection in the hospital. From the perspective of the distribution of Klebsiella pneumoniae in clinical departments, ICU is the department with a high detection rate of Klebsiella pneumoniae, accounting for 53.85%. This may be related to the prolonged hospitalization of ICU patients, critical condition, extensive use of antimicrobial drugs, frequent acceptance of invasive operations and other factors. Previous studies have reported that Klebsiella pneumoniae mainly infects the elderly over 70 years old.^20^ It is shown in this study that 40.38% of Klebsiella pneumoniae are isolated from populations over 70 years old. The reason may be that the elderly is prone to be infected with Klebsiella pneumoniae due to their old age, many underlying diseases, complex conditions and low immunity. Therefore, close observation should be given to susceptible populations, high-incidence areas, and high-risk parts of Klebsiella pneumoniae by medical staff, and populations infected with Klebsiella pneumoniae should be detected and reported in time, so as to effectively control nosocomial infection.

As indicated by the results of the drug sensitivity test, Klebsiella pneumoniae isolated from blood samples in our hospital has the highest drug resistance rate to ampicillin, up to 96.30%, and resistance rate >40% to piperacillin, cefazolin and cotrimoxazoleis, indicating that Klebsiella pneumoniae is highly resistant to the above-mentioned drugs. Consequently, the above drugs should not be used as clinical empirical drugs. Klebsiella pneumoniae is most sensitive to carbapenem antibacterial drugs such as meropenem, amikacin, levofloxacin, imipenem, and the drug resistance rate to the above four drugs are less than 10%, which is similar to the research results of Zhou R et al.^20^, suggesting that carbapenem antibiotics are preferred for empirical treatment of Klebsiella pneumoniae.

### Limitations of this study:

The number of subjects included in this study is limited, so the conclusions drawn may not be very convincing. In addition, we only analyzed the cases included in our hospital, which may not be representative enough. We look forward to a multi-center study in the future to reach more comprehensive conclusions.

## CONCLUSIONS

For patients with hypertensive intracerebral hemorrhage who have Klebsiella pneumoniae pulmonary infection after minimally invasive surgery, the pathogen of Klebsiella pneumoniae should be detected in time, and antibiotics should be taken rationally to effectively promote the elimination of cerebral hematoma in patients and protect the cranial nerve function of patients.

### Authors’ Contributions:

**WL & LX:** Designed this study, prepared this manuscript, and are responsible for the accuracy and integrity of the work.

**SZ:** Collected and analyzed clinical data.

**HZ:** Significantly revised this manuscript.
